# Toward Accurate Quantitative Elasticity Mapping of Rigid Nanomaterials by Atomic Force Microscopy: Effect of Acquisition Frequency, Loading Force, and Tip Geometry

**DOI:** 10.3390/nano8080616

**Published:** 2018-08-14

**Authors:** Guanghong Zeng, Kai Dirscherl, Jørgen Garnæs

**Affiliations:** DFM A/S (Danish National Metrology Institute), Kogle Alle 5, 2970 Hørsholm, Denmark; kdi@dfm.dk (K.D.); jg@dfm.dk (J.G.)

**Keywords:** elasticity mapping, rigid nanomaterials, atomic force microscopy, acquisition frequency, loading force, tip radius

## Abstract

Atomic force microscopy (AFM) has emerged as a popular tool for the mechanical mapping of soft nanomaterials due to its high spatial and force resolution. Its applications in rigid nanomaterials, however, have been underexplored. In this work, we studied elasticity mapping of common rigid materials by AFM, with a focus on factors that affect the accuracy of elasticity measurements. We demonstrated the advantages in speed and noise level by using high frequency mechanical mapping compared to the classical force volume mapping. We studied loading force dependency, and observed a consistent pattern on all materials, where measured elasticity increased with loading force before stabilizing. Tip radius was found to have a major impact on the accuracy of measured elasticity. The blunt tip with 200 nm radius measured elasticity with deviation from nominal values up to 13% in different materials, in contrast to 122% by the sharp tip with 40 nm radius. Plastic deformation is believed to be the major reason for this difference. Sharp tips, however, still hold advantages in resolution and imaging capability for nanomaterials.

## 1. Introduction

The demand for accurate measurement of mechanical properties of rigid materials with high spatial resolution has been driven by the rapid development of thin coatings, nanomaterials and nanocomposites. Probe-based indentation methods such as instrumented indentation testing (IIT) and atomic force microscopy (AFM) have been widely used to measure local mechanical responses [1,2]. Conventionally, IIT is the standard for mechanical testing of rigid materials, and well-established empirical models like Oliver and Pharr’s method have been widely adopted to extract both Young’s modulus and contact hardness from force-indentation curves [3,4]. AFM is a popular tool for testing soft materials due to its capability to work with small forces. Moreover, AFM can obtain high spatial resolution down to the nanoscale, which can be used to precisely locate the probe or examine samples before and after testing. This makes AFM a unique tool for mechanical testing of materials with nanoscale features. For example, AFM has been used to perform a miniaturized three-point bend test on metallic oxide nanowires [5,6], or nanoscale mechanical test on suspended graphene oxide monolayers [7].

AFM mechanical measurements can be performed in two distinctive modes. One can apply a relatively large loading force to induce elastic and plastic deformation. This mode is similar to IIT, where elasticity and plasticity can be evaluated at the same time from load-indentation curves. Alternatively, one can use a small loading force which only induces elastic deformation. The advantage of this method is that it is non-destructive, which enables high resolution mapping of mechanical properties of elasticity and adhesion. A classical version of AFM force mapping is force volume (FV) mapping. In FV, force spectroscopy is performed at each point on the map. During force spectroscopy, the AFM probe approaches the surface until a certain setpoint is reached before it retracts. A force-distance curve, which plots force vs. tip-sample distance, is obtained after proper calibration of the instrument. Force-distance curves are then analyzed in real-time to generate mechanical mapping. FV has existed for a long time, but its wide adoption has mainly been hindered by its low speed and consequently low resolution. In recent years, development in data acquisition and signal processing has allowed manufacturers to develop fast force mapping modes. These force mapping modes are given different brand names by manufacturers, such as PeakForce Quantitative Nanomechanical Mapping (PeakForce QNM^TM^, referred to as QNM for brevity, by Bruker), Quantitative Imaging (QI^TM^, by JPK Instruments), PinPoint^TM^ (by Park Systems), etc. These techniques follow the same principles of FV but use different methods to improve speed and signal, including probe driving mechanisms, signal processing, and data analysis [8]. Among these techniques, QNM is both the earliest and the most widely adopted one. Due to its ability to control small forces during imaging, QNM has been used to perform mechanical mapping of various soft materials [9,10], but QNM studies on rigid materials are still limited.

Accurate AFM measurement of elasticity is challenging due to many reasons, some of which are specific to rigid materials. Stiff AFM cantilevers are used for testing rigid materials, and determination of the spring constants of these cantilevers is not straightforward [11]. Calibration of deflection sensitivity can be a large source of uncertainty, especially at small deflections [12]. Flat surface and spherical tip geometry are assumed in the most popular Hertzian mechanical models (Hertzian, Derjaguin-Muller-Toporov (DMT) and Johnson-Kendall-Roberts (JKR)) [13], which are rarely fulfilled. This leads to error in measurements and indentation depth dependency. Furthermore, undesired lateral forces during AFM indentation causes buckling of cantilevers and sliding of tips, and eliminating these forces does not seem to be straightforward [14,15].

The purpose of the current study is to investigate the effect of different factors on the accuracy of elasticity mapping by AFM, with focus on rigid materials. We consider parameters such as acquisition frequency (FV vs. QNM), indentation depth, and tip radius, using several common rigid materials: fused silica (FS), highly oriented pyrolytic graphite (HOPG), Si(111) (Si) and Au(111) (Au). We investigated the dependence of measurements on indentation depth, starting with a very small indentation force. Based on this study, we make recommendations for the choice of indentation force and tip size for elasticity mapping of rigid materials at the nanoscale.

## 2. Materials and Methods

### 2.1. Materials

Sapphire, FS and HOPG were obtained as part of the Bruker PeakForce QNM sample kit (PFQNM-SMPKIT-12M) and Young’s modulus of the samples were used as reference values. Au(111) was epitaxially grown on cleaved mica with thickness of 150 nm (Molecular Imaging). Si(111) as 2″ *N*-type polished wafer was purchased from Sino-American Si Products Inc. (Hsinchu, Taiwan). Sapphire, FS, and Si were cleaned by sonication in ethanol, acetone, and water and drying with nitrogen. HOPG was freshly cleaved before measurements. Au was cleaned by 20 min UV-ozone treatment (ProCleaner by BioForce Nanosciences, Salt Lake City, UT, USA). TGT1 test grating (by NT-MDT Spectrum Instruments, Moscow, Russia), which consists of an array of sharp tips, was used as a tip characterizer. Tip characterization was performed by scanning with the TGT1 tip characterizer, followed by tip radius estimation by tip reconstruction using SPIP^TM^ (Image Metrology A/S, Hoersholm, Denmark) [16].

### 2.2. AFM Setup and Operation

AFM experiments were performed on Multimode 8 with PeakForce QNM^TM^ (Bruker Nano Surfaces, Santa Barbara, CA, USA). Diamond probes were used due to their high durability (up to certain force range). They can be cleaned by scanning on rough surfaces if contaminated during measurements. The consistency of diamond probes allows comparative measurements between samples. Two diamond probes (PDNISP-HS, Bruker Nano Surfaces, Santa Barbara, CA, USA) with spring constants of 360.9 and 395.4 N/m were used in this study. Both probes have corner-of-a-cube tip apex and a nominal tip radius of 40 nm, which was confirmed by tip characterization with TGT1. One probe was used as it is, which is referred to as the sharp probe. Another probe became blunt after prolonged use for indentation at high forces. The tip radius was increased from around 40 nm to around 200 nm. This probe is referred to as the blunt probe.

Spring constant from the manufacturer was used. Deflection sensitivity of the photodiode S, defined as *Z* piezo displacement divided by photodiode deflection, was calibrated on sapphire, assuming zero indentation with up to 0.3V deflection. FV was operated at 20 Hz (20 nm *Z* range, 781 nm/s velocity, 512 sampling points). QNM was performed at 2 kHz with PeakForce amplitude of 20 nm (40 nm *Z* range). This amplitude has been optimized for the force range 0.25–4 µN. It is small enough so that enough data points in the contact region are collected when force is low, and large enough so that deflection doesn’t exceed *Z* range when force is high.

QNM measurement started with calibration of tip radius R. This is done by QNM measurement on FS, a common reference material in the nanoindentation community. Tip radius was adjusted so that the calculated Young’s modulus matched the nominal value (Young’s modulus 72 GPa, reduced Young’s modulus with diamond 69.7 GPa). The estimated tip radius was then used for all subsequent measurements on other materials. To avoid the influence of instrument drift, calibration on FS was always performed immediately before or after measurements on other materials.

### 2.3. Data Analysis

Offline data analysis was done with MATLAB. Photodiode deflection vs. *Z* displacement curves were exported from FV or QNM raw data, and then converted to force-distance curves. Briefly, the baseline was corrected by subtraction with first-order linear fitting of the initial flat section. Photodiode deflection *D_pd_* was multiplied with deflection sensitivity *S* to obtain cantilever deflection *D*:

(1) $$D=D_{pd}\times S$$

To calculate tip-sample distance *d*, cantilever deflection *D* was subtracted from *Z* displacement:

(2) d=Z−D

Force *F* was calculated by multiplying cantilever deflection *D* and spring constant *k*:

(3) F=kD

Hertz model was used for calculating Young’s modulus from force-distance curves. The model assumes full elastic response. For spherical indentor, Hertz model gives the following relation between *F* and *d*:

(4) $$F=\frac{4}{3}E^{*}\times R^{\frac{1}{2}}{\left(d-d_{0}\right)}^{\frac{3}{2}}$$

where *R* is the tip radius, *d*_0_ is the contact point, and E^*^ is the reduced Young’s modulus of the sample with diamond indentor. E^*^ is related to the sample modulus *E* by:

(5) $$\frac{1}{E^{*}}=\frac{1-\nu_{i}^{2}}{E_{i}}+\frac{1-\nu^{2}}{E}$$

where *ν* is the Poisson’s ratio and subscript *i* indicates the indentor (diamond). All the Young’s moduli reported in the paper are the reduced Young’s modulus with diamond.

The retraction part of the force-distance curves was used for fitting. Fitting the curves to Equation (3) sometimes failed due to inhibited negative value of indentation (*d* − *d*_0_), even if *d*_0_ is set as fitting parameters. A more reliable equation for fitting is the linearized version:

(6) $$F^{\frac{2}{3}}={\left(\frac{4}{3}E^{*}\times R^{\frac{1}{2}}\right)}^{\frac{2}{3}}\left(d-d_{0}\right)$$

Combine Equations (1)–(3) and (6):

(7) $${\left(D_{pd}\times S\right)}^{\frac{2}{3}}={\left(\frac{\frac{4}{3}E^{*}\times R^{\frac{1}{2}}}{k}\right)}^{\frac{2}{3}}\left(Z-D_{pd}\times S-d_{0}\right)$$

From Equation (7) it can be seen that, because the outcome of the fit is the ratio between *D*^2/3^ and indentation (*d* − *d*_0_), any error of the spring constant *k* is factored in by the calibration of *R* from reference measurement on FS.

For QNM multi-channel images, deformation was determined at 15% of total height above the baseline, adhesion was found at the lowest point in the force-distance curve, and modulus is calculated from fitting with the DMT model, a variation of the Hertz model taking account into the adhesion force. The adhesion force *F_adh_* is added to the loading force *F* to give the effective loading force *F_eff_* for model fitting:

(8) $$F_{eff}=F+F_{adh}$$

where *F_adh_* is positive. *F_eff_* is then used as *F* in Equation (6) for model fitting. In all calculations (FV and QNM), the fit was made on the retraction curves with a lower limit of 10% and an upper limit of 90%.

## 3. Results and Discussion

### 3.1. Effect of Acquisition Frequency

**Figure 1:**
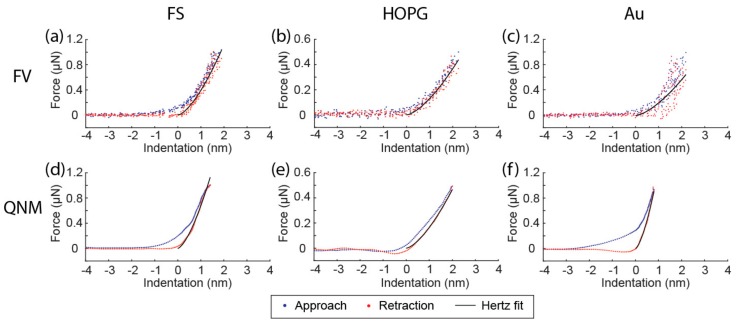
Force-indentation curves and Hertz fit of the retraction part from force volume (FV) (**a**–**c**) and PeakForce Quantitative Nanomechanical Mapping (QNM) (**d**–**f**) recorded on fused silica (FS), highly oriented pyrolytic graphite (HOPG), and Au(111) (Au). Position of zero indentation was determined from the Hertz fit and does not necessarily indicate the contact point.

Force-indentation curves from low frequency mode FV (20 Hz) and high frequency mode QNM (2 kHz) performed with the sharp probe were plotted and fitted to the Hertz model (Figure 1). The noise level is much lower in QNM compared to FV. The baseline noise floor is around 20 nN and 80 nN for QNM and FV, respectively. A more striking difference is noticed when curves from QNM and FV on Au are compared (Figure 1c,f). The noise level in the contact region of FV on Au is so large that it is difficult to define the zero indentation point (Figure 1c). In contrast, the noise level of QNM on Au is much smaller. In AFM force spectroscopy, the noise is dominated by the thermal noise. The much lower noise level in QNM is presumably due to averaging and possible signal filtering [8]. While time-dependent mechanical response has been frequently observed on viscoelastic materials such as polymers [17] and biological materials [8], further investigation is needed to fully explain the somewhat peculiar behavior of Au. It can be speculated that the observed frequency dependency may be linked to the relatively high plasticity of Au. The high plasticity of gold is evident from the large hysteresis of the force indentation curves from Au (Figure 1c,f). Exactly how this property has caused the high noise level at low frequency indentation in Figure 1c is puzzling, as Au is not known to be viscoelastic.

**Figure 2:**
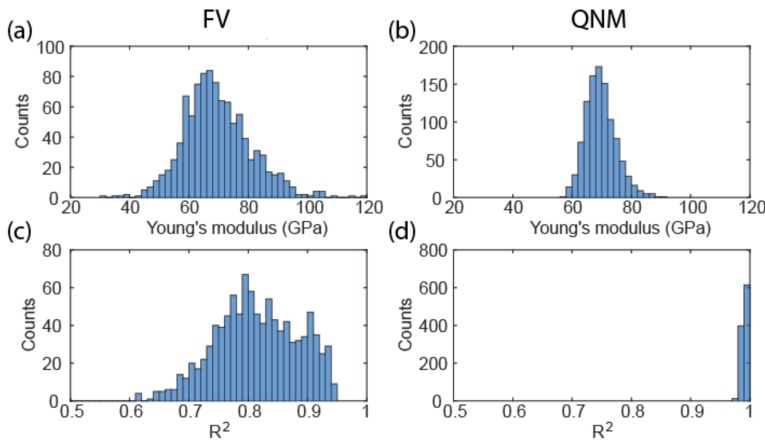
Histograms showing the distribution of Young’s modulus of FS determined from the Hertz fit of 1000 force-indentation curves from FV (**a**) and QNM (**b**). Corresponding coefficient of determination of the fit are shown in (**c**,**d**).

As expected, the lower noise level of QNM compared to FV resulted in a much narrower distribution of Young’s modulus fitted from the data (Figure 2). While both data follow Gaussian distribution, the spread of FV is much larger than that of QNM, with a standard deviation of 12.3 GPa compared to the 4.6 GPa of QNM. The goodness-of-fit of FV indicated by R^2^ is much worse than that of QNM as a result of high noise in the contact region. Assuming the above standard deviation as population values, the sampling numbers required for achieving a 5% error margin at 95% confidence level are 49 and seven for FV and QNM, respectively. This means that reliable measurement can be done with a smaller number of samples for QNM. Overall, also taking into account the higher acquisition frequency, QNM is a much faster technique than FV.

### 3.2. Effect of Loading Force and Tip Geometry

**Figure 3:**
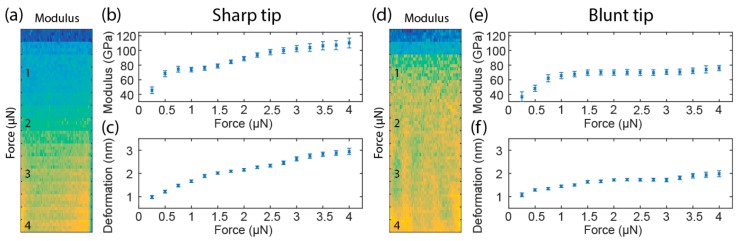
Young’s modulus map (**a**), Young’s modulus plot (**b**) and deformation plot (**c**) with increasing loading force from QNM measurement of the sharp tip on FS; (**d**–**f**) Corresponding map and plots from the blunt tip. Maps were recorded on a 400 nm square at 64 × 64 resolution, and loading force was increased from top to bottom from 0.25 to 4 µN with a 0.25 µN step between every four lines. Modulus and deformation plots were extracted from maps. Each modulus data point is calculated from 192 (3 × 64, transition line skipped) points. Error bars represent standard deviation.

Loading force or indentation depth dependence is one of the major issues in achieving accurate mechanical measurements with AFM. The low noise level of QNM makes it possible to measure with very small forces, which allows us to investigate the effect of loading force, starting from very small forces. QNM measurements were performed on FS with varying loading force (Figure 3). When a sharp tip with around 40 nm radius was used, the measured Young’s modulus shows a three-stage evolution with increasing force (Figure 3b). The Young’s modulus rises sharply as force increases from 0.25 to 0.75 µN in stage one, stabilizes until the force reaches 1.5 µN in stage two, and then rises again until the force reaches 4 µN in stage three. In the later part of stage three (2.5–4 µN), the deviation of data increased. This is visualized on the mechanical map, where the modulus begins to fluctuate, particularly near the edge (Figure 3a). A possible cause of this fluctuation at the edge is significant plastic deformation, which begins to affect neighboring pixels. The edge pixels do not have neighboring pixels on both sides, so their moduli are different, causing this edge effect (yellow and blue pixels on the lower edge of Figure 1a). This is also supported by the observation that the approach and retraction part of the force-indentation curves begin to separate at high forces (data not shown). In contrast, when a blunt tip with around 200 nm radius was used, only stage one and two were observed (Figure 3e), and the onset of stage two is shifted to larger force. As expected, deformation is smaller compared to the sharp tip (Figure 3c,f), and plastic deformation seems to be absent.

**Figure 4:**
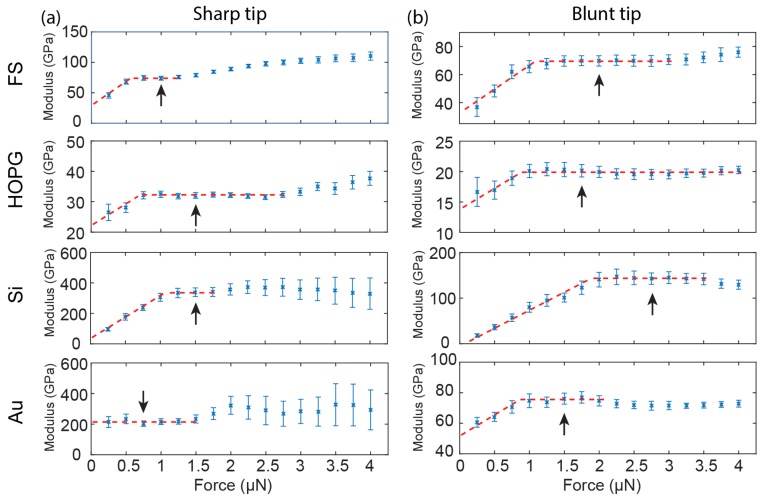
Young’s modulus plots at 0.25–4 µN loading forces from QNM measurements on FS, HOPG, Si(111) (Si), and Au with the sharp tip (**a**) and the blunt tip (**b**). Dashed red lines are added to visualize the trend of modulus variation with increasing loading forces. Arrows indicates the data points used for reporting reduced Young’s modulus.

We extended the measurements to HOPG, Si, and Au to check whether the observations on FS were material specific (Figure 4). Similar trends are observed in all samples with the exception of the sharp tip on Au. For the sharp tip, the measured Young’s modulus rises with increasing loading forces and stabilizes, before entering the plastic range indicated by the expanded error bars of the data. For the blunt tip, the measured Young’s modulus rises and stabilizes with increasing loading forces.

**Figure 5:**
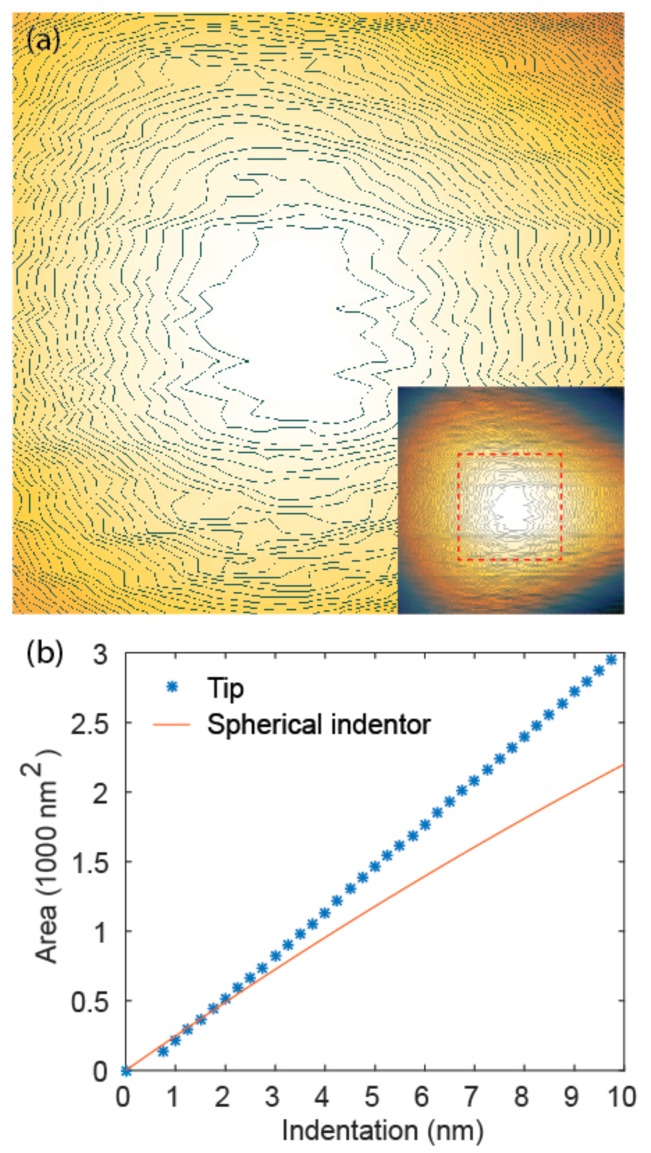
(**a**) Atomic force microscopy (AFM) height image of the apex of the sharp tip with contour lines, height step 0.25 nm, obtained by scanning on TGT1. Inset shows the whole tip; (**b**) Area of the tip contours at 0–10 nm tip height determined from AFM image, compared with expected contour area from a spherical indentor with the effective tip radius calibrated on FS (40 nm).

If we assume that plastic deformation can cause overestimation of Young’s modulus at large forces, it requires further explanation that even within the elastic range, i.e., at small forces, the measured modulus increased with loading force (stage one, Figure 3b,e). Potential causes of this phenomenon could be surface contamination, asperity on the tip apex, or different mechanical properties of materials near the surface. Surface contamination can largely be excluded because similar results were obtained on fresh surfaces and tips. Asperity on the tip apex could have influences on mechanical measurements at small indentations. Local protrusions, for example, can cause overestimation of the tip radius, leading to underestimation of modulus according to Equation (6). To verify this, we measured the height profile of the sharp tip by scanning on a tip characterization sample TGT1 and calculating the area of the contours at different heights (Figure 5a). The area of the contours at different indentation depths (height from the apex) was then compared with that expected from a sphere (Figure 5b). While measurement of the tip profile is subject to system instability at this scale, and therefore not perfectly accurate, the comparison suggests that at ultra-small indentations (<2 nm), tip radius tends to be overestimated and therefore Young’s modulus is underestimated. At large indentations, the trend reverses, leading to overestimation of the Young’s modulus. This observation, together with plastic deformation, serves as a plausible explanation for the observed dependency of Young’s modulus on loading force (Figure 3b), where the modulus rises, stabilizes, and then rises again with increasing loading force. An alternative/additional explanation of the low Young’s modulus at small forces could be surface water. To sum up, the indentation depth dependent behavior of Young’s modulus measurement can be largely explained by a combination of plastic response and non-ideal tip geometry.

Technically, the stabilization of the measured Young’s modulus after the initial increase allows us to determine the loading force for reporting Young’s modulus (Figure 4, arrows). These plateaus in the modulus vs. loading force curves represent the range where both surface effect and plastic response are negligible. This procedure can be done for both the sharp tip and the blunt tip, although the plateau is narrower for the sharp tip due to early onset of plastic deformation. However, the reported values of Young’s moduli from the sharp tip and the blunt tip are dramatically different (Table 1). Deviation of the reduced Young’s moduli from reference values ranges from 69% to 122% for the sharp tip, compared with −6% to 13% for the blunt tip (Table 1). In all cases, the sharp tip significantly overestimates the Young’s modulus, whereas accuracy of the blunt tip is much higher.

**Figure 6:**
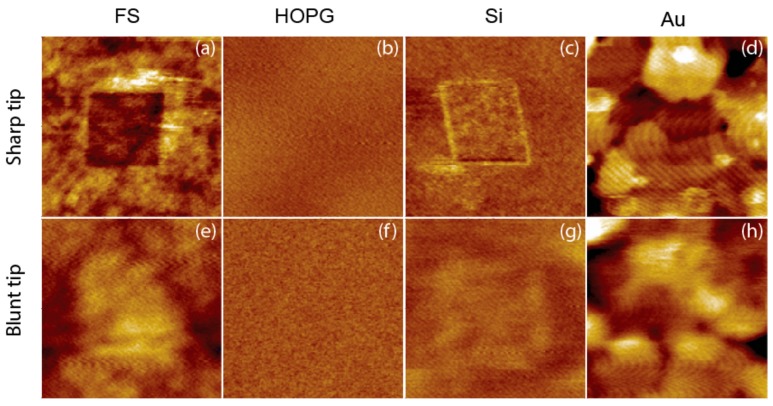
1 × 1 µm AFM height images of materials recorded after QNM measurements in 400 nm squares with sharp and blunt tips, using the loading forces specified in Figure 5 for each tip and material combination. (**a**–**d**) are images from the sharp tip, and (**e**–**h**) are images from the blunt tip. Resolution is 128 × 128, Z range is 4 nm for Au and 2 nm for FS, HOPG and Si.

The large overestimation of Young’s modulus by the sharp tip even at small forces suggests that significant plastic deformation could have been induced by the high stress associated with a small contact area. Note that the radius of the sharp tip is approximately one fifth of that of the blunt tip, which translates into 25 times as large stress with the same loading force. This hypothesis is confirmed by examining the surface after QNM measurements (Figure 6). With the exception of HOPG, the sharp tip induced permanent deformation on all other materials (deformation on Au is obscured by surface features, but the right and bottom borders are clear), whereas the blunt tip hardly left any indents after QNM measurements. It has to be noted that the images were recorded with the same tip for QNM, which explains the different lateral resolution of the images from sharp and blunt tips. However, vertical resolution, which addresses the surface deformation, is largely independent of tip size. A similar tip radius effect has been observed for soft materials [21]. High stress associated with the sharp tip was also believed to have caused the so-called “skin-effect”, i.e., overestimation of Young’s modulus at the surface. Similarly, this effect can be eliminated by using blunt tips [21].

It is noteworthy to point out that, apart from the factors investigated so far, there are several other factors and assumptions that affect the accuracy of the measurements. Calibration of deflection sensitivity *S* could be complicated by indentation in the sapphire, which may not be negligible for stiff cantilevers. We have used reference measurements on FS to calibrate the tip radius. This method has the advantage of eliminating the need for accurate spring constant calibration. However, it is based on the assumptions of the Hertz model: (1) tip apex is spherical; (2) deformation is fully elastic; (3) surface is flat. In reality, it is unlikely that these conditions can be fulfilled at the same time. For example, at small forces, deformation is more likely to be elastic, but defects at the tip apex have a larger influence on the tip shape. Furthermore, tip rotation, a problem specific to AFM, is likely to have an impact. As already mentioned briefly in the introduction, due to geometric constraints of the cantilever-based probe, the tip must rotate and move laterally during indentation. This will cause buckling of the cantilever, which leads to error in force measurement. In addition, the tip might rotate and slide on the surface. This effect is more evident at large cantilever deflections and high sample moduli [15]. The tip rotation effect may also partially explain the larger error from using the sharp tip compared with the blunt tip. The sharp tip is more likely to dig into the sample and get stuck, whereas the blunt probe could slide on the sample. The latter causes less cantilever buckling. This explanation is speculative however, due to the fact that it is difficult to isolate the tip rotation effect from other factors.

### 3.3. Imaging Capabilities

**Figure 7:**
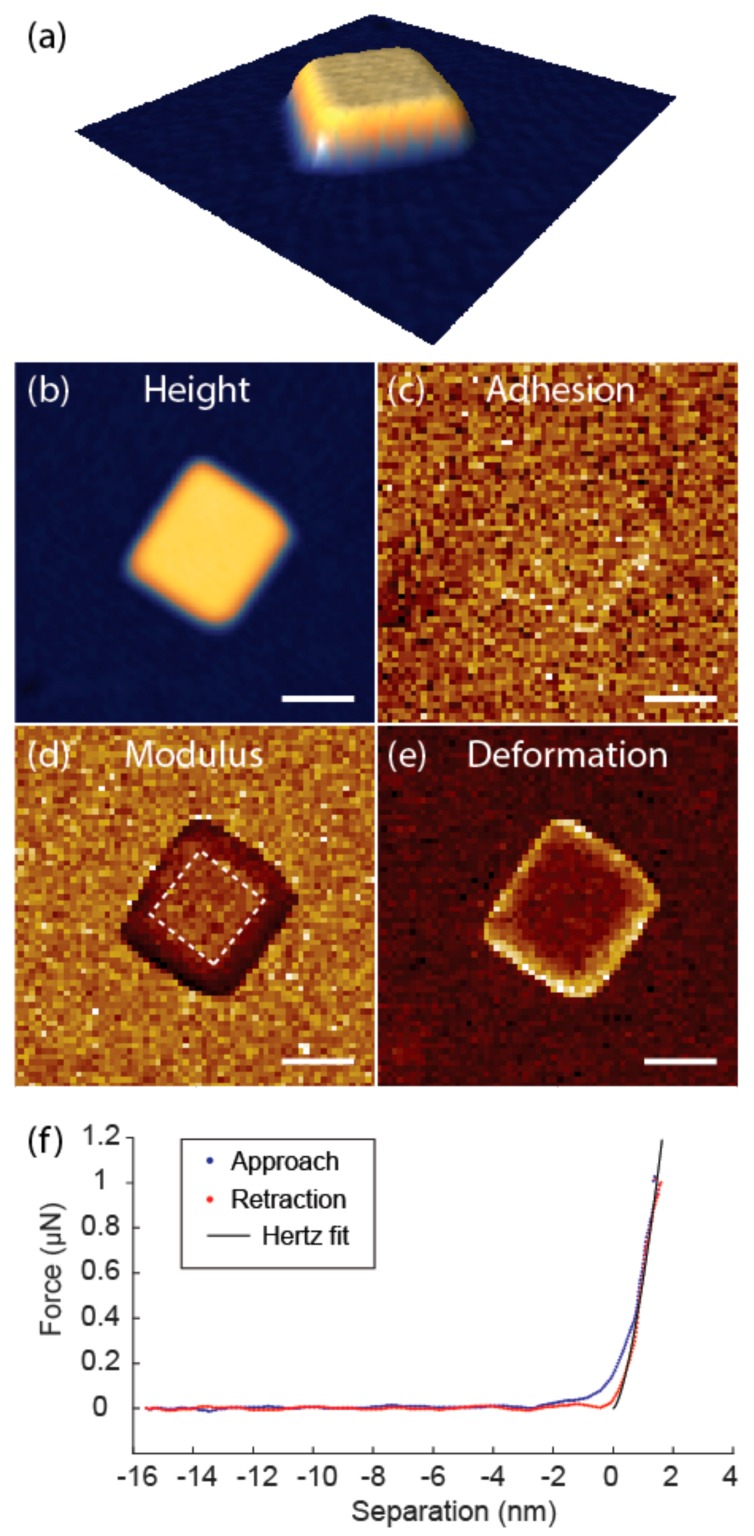
Height (**a**,**b**), adhesion (**c**), Young’s modulus (**d**), and deformation (**e**) images from QNM measurement on a TiO_2_ nanocrystal. Height range 12 nm, adhesion range 60 nN, modulus range 200 GPa, deformation range 1.8 nm, resolution 64 × 64, scale bar = 100 nm. Young’s modulus was calculated from the area indicated by dashed lines; (**f**) Force-indentation curve from QNM on the nanocrystal and Hertz fit of the retraction part.

The blunt tip holds advantages over the sharp tip for accurate quantitative measurements. However, this comes at the cost of imaging capabilities. We measured TiO_2_ nanocrystals with a lateral dimension of 100–200 nm using both the sharp and the blunt tip, and only the sharp tip was able to resolve the morphology of the nanocrystals (Figure 7). It was also observed that the nanocrystals were more likely to be detached or dislocated by the blunt tip during scanning, probably due to large interaction forces with the blunt tip. The thickness of the nanocrystal is 10.5 nm (Figure 7a,b), which is sufficiently large compared to the small deformation of 0.9 nm (Figure 7e, results from the deformation image, which tend to be lower than the actual indentation. Offline analysis from the force curves gives an indentation depth of 1.8 nm, which is still small compared to sample thickness (less than 20%)). This means that the possibility of the substrate effect for mechanical measurements can be excluded. From the mechanical mapping, we can also obtain multidimensional information. The adhesion force from the adhesion image is around 50 nN (Figure 7c), which lies within the range of the noise floor (Figure 7f). This indicates that adhesion of the TiO_2_ nanocrystal to the tip is low and cannot be accurately measured with this probe. By comparing the Young’s modulus map to the height image, it could be seen that moduli measured on the edge are lower than that on the top due to the inclined edge plane. We should therefore exclude the edge for the calculation of Young’s modulus. The measured Young’s modulus of the TiO_2_ nanocrystal from the top plane is 109.4 ± 16.7 GPa, which agrees reasonably well with the 140 GPa measurement for anatase crystalline film measured by IIT [22].

To achieve accurate elasticity mapping of rigid materials, it is recommended to work in high frequency mechanical mapping mode, starting with small loading forces and increasing the loading force in small steps until the first plateau is reached. Blunt tips are preferred for good accuracy. The trade-off is that their imaging performance and spatial resolution is not as good as sharp tips.

## 4. Conclusions

We have investigated the effect of acquisition frequency, loading force, and tip geometry on elasticity mapping of common rigid materials. High frequency mode offers fast speed and low noise level, and therefore enables small loading force. A sharp tip with 40 nm radius and a blunt tip with 200 nm radius were compared. The measured elasticity increased with increasing loading force, stabilized with both tips, and then increased again only for the sharp tip. When the blunt tip was used, the deviation of elasticity from nominal values was within 13%. In contrast, the sharp tip resulted in deviation up to 122%. Plastic deformation is proposed to be the main reason for inaccurate measurement by the sharp tip, and other possible factors have been discussed. It is recommended that sharp tips be used for better imaging capability and resolution, and blunt tips be used when good accuracy of elasticity is demanded.

## Figures and Tables

**Table 1:** Young’s moduli (E) and reduced Young’s modulus (E*) of FS, HOPG, Si, and Au measured with sharp and blunt tips and deviation from reference values in percentage (**Δ%**). Data points are reported as the mean (standard deviation) from three independent measurements, reported from the loading force indicated with arrows in Figure 5.

				Sharp Tip		Blunt Tip	
Sample	E/GPa	Poisson’s Ratio	E*/GPa	E*/GPa	Δ%	E*/GPa	Δ%
**FS**	72.0 ^a^	0.17	69.7	69.7 (0.4)	-	69.7 (3.5)	-
**HOPG**	18.0 ^b^	0.20	18.4	31.1 (1.0)	69	20.8 (1.4)	13
**Si(111)**	168.9 ^c^	0.26	156.8	347.6 (7.6)	122	147.2 (10.3)	−6
**Au(111)**	78.5 ^d^	0.42	88.0	179.8 (21.6)	104	88.2 (1.4)	0

^a^ Ref. [3], ^b^ Ref. [18], ^c^ Ref. [19], ^d^ Ref. [20].
